# Telehealth amid the COVID19 pandemic: perception among Asian, Native Hawaiian and Pacific Islander cancer patients

**DOI:** 10.2217/fon-2021-0136

**Published:** 2021-06-09

**Authors:** Michael Meno, Justin Abe, Jami Fukui, Christa Braun-Inglis, Ian Pagano, Jared Acoba

**Affiliations:** ^1^Department of Medicine, John A. Burns School of Medicine, University of Hawaii, Honolulu, HI 96813, Hawaii; ^2^University of Southern California, Los Angeles, CA 90007, USA; ^3^University of Hawaii Cancer Center, Honolulu, HI 96813, Hawaii; ^4^Queen's Medical Center, Honolulu, HI 96813, Hawaii

**Keywords:** COVID-19, health disparity, pandemic, patient satisfaction, race, telehealth, telemedicine, teleoncology

## Abstract

**Aim:** To assess the perception of telehealth visits among a multiracial cancer population during the coronavirus disease 2019 pandemic. **Methods:** This cross-sectional study was conducted at outpatient cancer clinics in Hawaii between March and August 2020. Patients were invited to participate in the survey either by phone or email. **Results:** Of the 212 survey respondents, 61.3% were Asian, 23.6% were White and 15.1% were Native Hawaiians or Pacific Islanders. Asians, Native Hawaiians and Pacific Islanders were less likely to desire future telehealth visits compared with Whites. Predictors with regard to preferring future telehealth visits included lower income and hematopoietic cancers. **Conclusion:** The authors found racial differences in preference for telehealth. Future studies aimed at overcoming these racial disparities are needed to provide equitable oncology care.

The impact of the COVID-19 pandemic has accelerated the development of new models of care in oncology practice. Individuals with cancer are at an increased risk of mortality from COVID-19, leading to a greater need for precautions, such as social distancing [1,2]. In an attempt to decrease in-person office visits, oncology clinics have altered treatment schedules by increasing intervals between treatments [3,4]. The American Society of Clinical Oncology recommended adoption of telemedicine for patients not requiring a physical exam, treatment or in-office diagnostic testing [5]. Furthermore, the Department of Health and Human Services lifted restrictions, allowing wider adoption of telehealth visits as a substitution for in-person visits without diminishing reimbursements [6]. As a result, oncologists have rapidly adopted the use of telehealth in place of the traditional office visit to decrease the risk of transmitting the virus among patients and providers [7,8].

In general, telehealth refers to the use of telemedicine (defined by the Centers for Medicare and Medicaid Services as real-time interactive audio and video telecommunication) and/or telephone visits [9]. As a modality of provider-to-patient interaction, telehealth is relatively young, existing since only the early 1990s. Prior to the COVID-19 pandemic, telehealth was traditionally used for the delivery of healthcare services where distance was a critical factor. At present, the majority of patients are being converted to telehealth visits because of COVID-19 directives rather than distance. Recent teleoncology studies have demonstrated high rates of satisfaction with telehealth during the COVID-19 pandemic [10–12]. However, teleoncology has not been extensively studied in Asian and indigenous Pacific Islander populations, such as Native Hawaiians, who may have different experiences with the modality [13]. This study aimed to assess the perception of telehealth visits among a multiracial cancer population for whom in-person visits were the standard of care prior to COVID-19.

## Methods

### Participants & eligibility criteria

This study was conducted at outpatient cancer clinics in Hawaii affiliated with the Queen's Health Systems and Hawaii Pacific Health. Together, these clinics care for about 70% of all cancer patients in the state. Patients who completed a telehealth visit between March and August 2020 were eligible to participate. Adults aged 18 years and older, with any cancer type and treatment intent, were eligible. Participants needed to be literate in English.

### Data collection & measurement

Participants were approached sequentially in the survey time frame. Patients were invited to participate in the survey either by phone or email. All surveys were completed anonymously, and no personal health information or personally identifiable information was collected.

Demographic data collected included sex, age, education level, income, insurance type, race, type of cancer and stage of cancer. The authors developed a survey using Likert-type scales to evaluate patients' telehealth experience. Patients also rated their telehealth visit in comparison with a traditional face-to-face office visit (office visit is better, telehealth visit is better or no difference). Survey questions were adapted from a study by Donelan *et al.*, who published their assessment of the Massachusetts General Hospital telehealth experience [14]. A final open-ended question allowed participants to offer feedback on issues not covered in the survey.

Age was categorized as younger than 60, 60–79 and 80 and older. Education level was grouped into three categories: up to some college but no formal degree, associate or bachelor's degree and master's or doctoral degree. Categories for income included prefer not to say, <$30,000 per year, $30,000–89,999 per year and $90,000 or more per year. Insurance was categorized as private insurance; Medicare with a supplement; and other insurance, which included Medicaid and Medicare without a supplement. Patients self-identified their race and were grouped as either White or Native Hawaiian or Pacific Islander or Asian. Cancer type was grouped as gastrointestinal, hematopoietic (acute myeloid leukemia, myelodysplastic syndrome, lymphoma or myeloma), genitourinary, breast and lung or other. Cancer stage was grouped as ‘I do not remember,’ stage 0–2 and stage 3–4.

The primary end point of the study was the determination of a patient's perception of the overall quality of her or his telehealth visit. The secondary end point was establishment of the preference for future visits to be via telehealth compared with the traditional office visit. This study was also designed to determine the degree to which patient demographics and cancer type impacted these outcomes.

### Statistical methods

Nonparametric descriptive statistics were used to evaluate characteristics of standard demographic data, tabulated by method of telehealth visit. A p < 0.05 was considered statistically significant. Overall quality was analyzed by comparing patients who preferred telehealth or found no difference between telehealth and office visits with patients who preferred office visits. Analysis of the desire for future visits compared patients who agreed with having future visits via telehealth with those who were neutral or disagreed with having future telehealth visits. Logistic regression models for quality of the telehealth visit and desire for future telehealth visits were built to obtain odds ratio (OR) and 95% CI. Multivariate models were adjusted for age, sex, race, insurance status, education level, distance from the oncology office, income, cancer type and stage and inclusion of video. Statistical analyses were performed with SPSS Statistics 27.0 (IBM Corporation, NY, USA).

### Ethics

Approval for this study was granted by the Queen's Medical Center Research and Institutional Review Committee, the Hawaii Pacific Health Institutional Review Board and the Western Institutional Review Board. In addition, informed consent was obtained from the participants involved.

## Results

A total of 450 patients were contacted, and 224 patients completed the survey, for a response rate of 49.8%. Patients were excluded from analysis if they stated ‘prefer not to say’ for the following demographics: race, age and distance. In addition, patients (n = 5) were excluded if their race could not be categorized as White, Native Hawaiian or Pacific Islander or Asian. A total of 212 patient surveys were included in the final analysis.

Of the 212 survey respondents, 138 (65.1%) were female and 74 (34.9%) were male (Table 1). The majority of participants were Asian (130; 61.3%), followed by White (50; 23.6%) and Native Hawaiian or Pacific Islander (32; 15.1%). The most common cancer type was breast cancer (43.4%), followed by gastrointestinal cancer (23.1%) and hematopoietic cancer (13.2%). Most telehealth visits included video (65.5%). Of the video platforms used, the most common was FaceTime (33.7%), followed by MyChart (28.3%), Zoom (24.1%), Doximity (12.7%) and Webex (1.1%). A large fraction of the patients did not remember which platform was used (27.8%).

**Table 1.** Patient characteristics.

Characteristic	All patients		Audio only		Audio and video		p-value
	n	%	n	%	n	%	
**Overall**	212	100	73	34.4	139	65.6	
**Sex**							0.09
Female	138	65.1	42	57.5	96	69.1	
Male	74	34.9	31	42.5	43	30.9	
**Age, years**							0.23
<60	62	29.2	16	21.9	46	33.1	
60–79	128	60.4	49	67.1	79	56.8	
≥80	22	10.4	8	11.0	14	10.1	
**Education**							0.95
Less than associate	76	35.8	27	37.0	49	35.3	
Associate or bachelor's	105	49.5	36	49.3	69	49.6	
Master's or doctorate	31	14.6	10	13.7	21	15.1	
**Income**							**0.03**
Prefer not to say	40	18.9	12	16.4	28	20.1	
<$30,000	35	16.5	18	24.7	17	12.2	
$30,000–89,999	70	33.0	27	37.0	43	30.9	
≥$90,000	67	31.6	16	21.9	51	36.7	
**Insurance**							0.91
Medicare without a supplement, Medicaid, other	38	17.9	12	16.4	26	18.7	
Medicare with a supplement	73	34.4	26	35.6	47	33.8	
Private	101	47.6	35	47.9	66	47.5	
**Ethnicity/race**							0.14
Asian	130	61.3	51	69.9	79	56.8	
Native Hawaiian or Pacific Islander	32	15.1	7	9.6	25	18.0	
White	50	23.6	15	20.5	35	25.2	
**Distance**							0.06
15-min drive or less	63	29.7	26	35.6	37	26.6	
16–30-min drive	80	37.7	32	43.8	48	34.5	
More than 30-min drive	39	18.4	8	11.0	31	22.3	
Flight	30	14.2	7	9.6	23	16.5	
**Cancer type**							**0.045**
Gastrointestinal	49	23.1	23	31.5	26	18.7	
Hematopoietic	28	13.2	5	6.8	23	16.5	
Genitourinary	20	9.4	10	13.7	10	7.2	
Lung and other	23	10.8	7	9.6	16	11.5	
Breast	92	43.4	28	38.4	64	46.0	
**Cancer stage**							0.54
I do not remember	45	21.2	18	24.7	27	19.4	
Stage 0–2	97	45.8	34	46.6	63	45.3	
Stage 3–4	70	33.0	21	28.8	49	35.3	
**Overall quality**							**0.007**
Telehealth is better or no difference	139	65.6	39	53.4	100	71.9	
Office visit is better	73	34.4	34	46.6	39	28.1	
**Preference for future telehealth visits**							0.28
Agree	121	57.1	38	52.1	83	59.7	
Neutral or disagree	91	42.9	35	47.9	56	40.3	

Bold values denote statistical significance at the p < 0.05 level.

Characteristics were similar between patients who had telehealth visits that included video and those who had audio-only visits; however, patients with higher income were more likely to have a visit that included video (p = 0.03). Patients who experienced video visits perceived the telehealth visit as being better or no different from the traditional office visit more often than patients with audio-only visits (p = 0.007).

**Figure 1.**
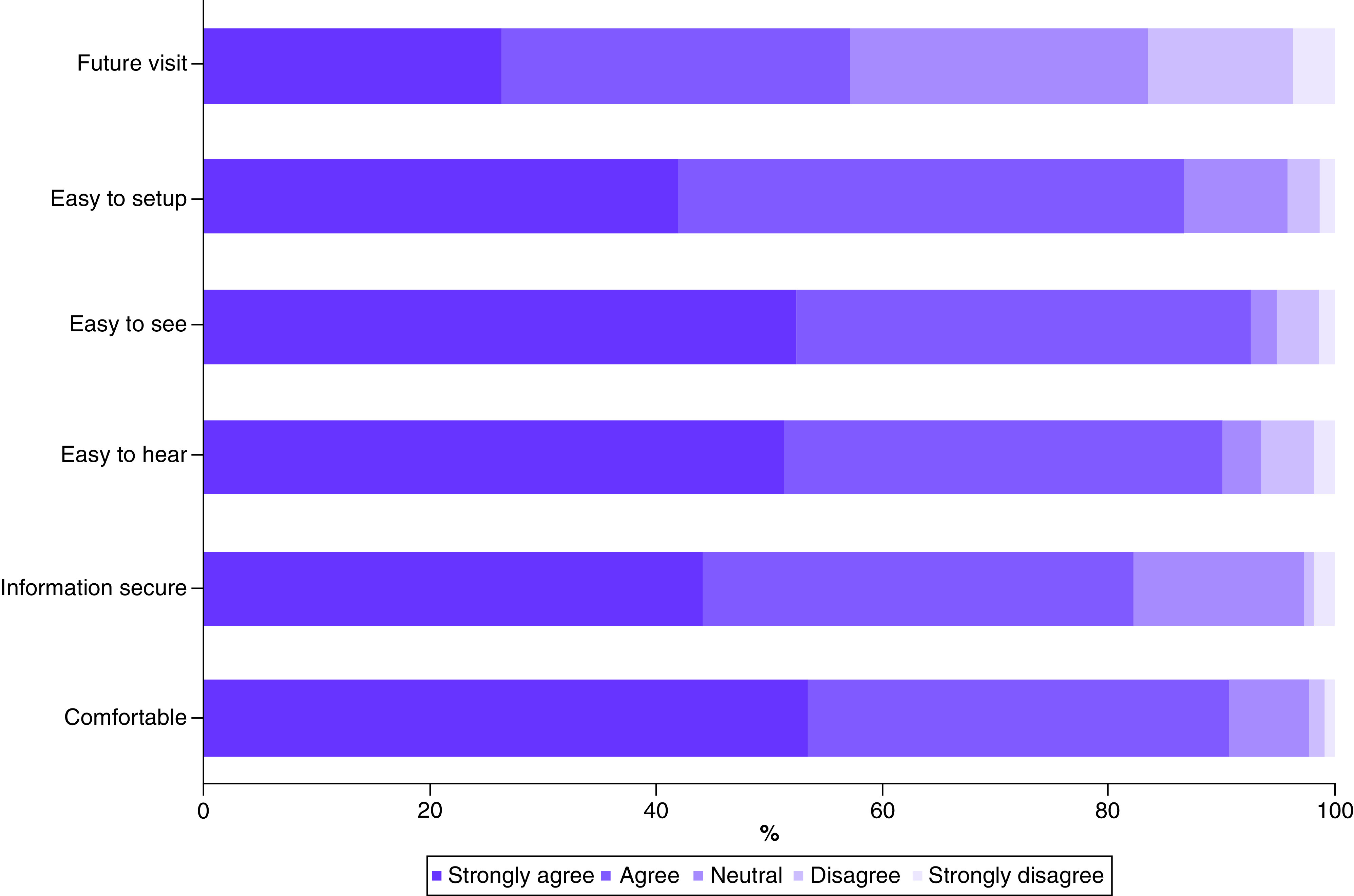
Patient experiences of the telehealth visit.

Based on Likert scale questions, patients' experiences with telehealth were mostly positive (Figure 1). Over 90% of patients were comfortable with the telehealth visit and had no difficulties seeing or hearing the physician. The majority of patients were satisfied with the technology aspect of the visit, with 86.8% agreeing or strongly agreeing that it was easy to set up the telehealth visit and 82.1% agreeing or strongly agreeing that their information was securely transmitted. However, when asked if they would like future visits to be telehealth, only 57.1% of respondents agreed or strongly agreed, whereas 26.4% were neutral and the remaining 16.5% disagreed or strongly disagreed.

The items were asked in the survey as followed – future visit: ‘I would like some of my future visits to be telehealth visits rather than face-to-face visits’; easy to set up: ‘it was easy to set up my telehealth visit using my phone/computer/tablet’; easy to see: ‘it was easy to see my doctor during the telehealth visit (video visit only)’; easy to hear: ‘it was easy to hear my doctor during the telehealth visit’; information secure: ‘my information was securely transmitted during my telehealth visit’; comfortable: ‘I felt very comfortable with my telehealth visit’.

**Figure 2.**
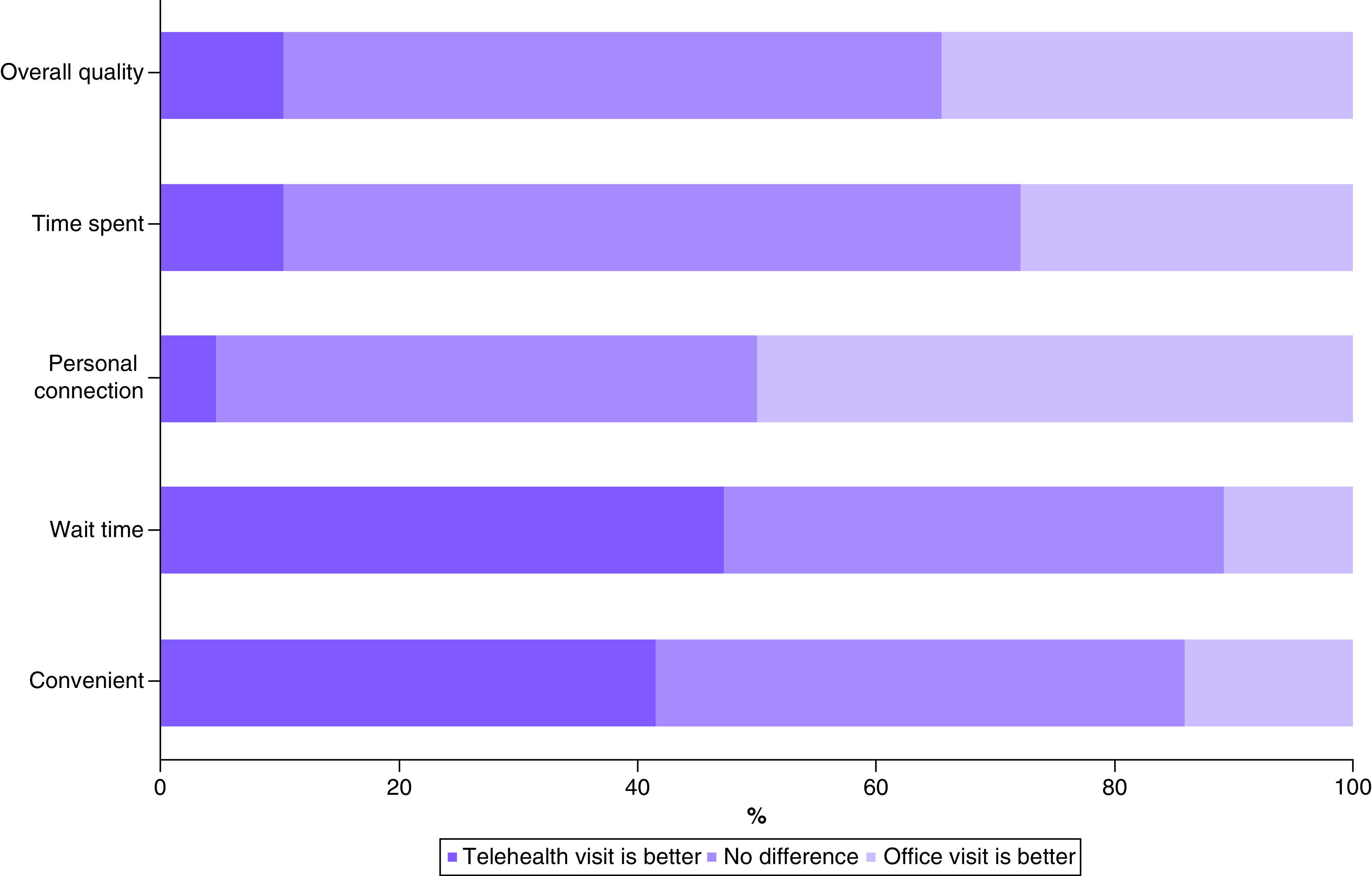
Patient-reported preferences for telehealth visits compared with office visits.

Most patients felt that the overall quality of the telehealth visit was the same as that experienced with an office visit (55.2%) or better (10.4%). Conversely, 34.4% of patients felt that the overall quality of office visits was better (Figure 2). Patients favored telehealth or felt a telehealth visit was similar to an office visit with regard to time spent with the provider (72.2%), wait time (89.2%) and finding a convenient time for the visit (85.8%). However, when asked about the personal connection they felt with their provider, half (50.0%) of the patients found the personal connection in office visits to be better.

The items were asked in the survey as followed - Overall quality: ‘overall quality of the visit’; time spent: ‘amount of time I spent with the doctor’; personal connection: ‘personal connection I feel with the doctor during the visit’; wait time: ‘amount of time I wait for the doctor’; convenient: ‘finding a convenient time for the visit’.

Logistic regression models were created to identify predictors of overall quality of the telehealth visit and the desire to have future telehealth visits. The only predictor of visit quality was the inclusion of video, which was significantly associated in both univariate (OR: 2.24; 95% CI: 1.24–4.03) and multivariate (OR: 2.22; 95% CI: 1.12–4.38) analyses. No other predictors of visit quality were identified.

There were several significant predictors of the desire for future telehealth visits (Table 2). On univariate analysis, patients with hematopoietic cancers (OR: 2.87; 95% CI: 1.11–7.41) and those who favorably rated the quality of the visit (OR: 8.74; 95% CI: 4.55–16.81) were more likely to want a future telehealth visit. Conversely, Asians (OR: 0.34; 95% CI: 0.16–0.70) and Native Hawaiians and Pacific islanders (OR: 0.32; 95% CI: 0.12–0.82) were less likely to desire future telehealth visits compared with Whites. These factors remained significant on multivariate analysis. In addition, lower income of $30,000–89,999 was associated with the desire for future telehealth visits compared with income ≥$90,000 (OR: 3.85; 95% CI: 1.44–10.30). Video during the telehealth visit was not a significant variable with regard to wanting future visits to be telehealth.

**Table 2.** Univariate and multivariate linear regression for variables predicting preference for future telehealth visits.

Factor	Univariate		Multivariate	
	Adjusted OR (95% CI)	p-value	Adjusted OR (95% CI)	p-value
**Female sex**	0.73 (0.41–1.29)	0.27	0.69 (0.24–1.92)	0.47
**Age, years, <60 as reference**				
60–79	0.90 (0.48–1.66)	0.73	0.38 (0.09–1.61)	0.19
≥80	0.68 (0.25–1.80)	0.43	0.78 (0.33–1.85)	0.57
**Education, master's or doctorate as reference**				
Less than associate	0.76 (0.32–1.80)	0.53	0.52 (0.15–1.83)	0.31
Associate or bachelor's	0.65 (0.29–1.50)	0.31	0.63 (0.18–2.16)	0.46
**Income, ≥$90,000 as reference**				
Prefer not to say	0.78 (0.36–1.71)	0.53	0.81 (0.29–2.28)	0.68
<$30,000	1.29 (0.56–2.96)	0.55	2.63 (0.79–8.72)	0.12
$30,000–89,999	1.55 (0.78–3.08)	0.21	**3.85 (1.44–10.30)**	**0.007**
**Insurance, private as reference**				
Medicare without a supplement, Medicaid, other	1.03 (0.49–2.19)	0.93	0.77 (0.26–2.31)	0.65
Medicare with a supplement	1.34 (0.73–2.48)	0.34	1.16 (0.49–2.71)	0.74
**Ethnicity, White as reference**				
Asian	**0.34 (0.16–0.70)**	**0.004**	**0.26 (0.10–0.71)**	**0.01**
Native Hawaiian or Pacific Islander	**0.32 (0.12–0.82)**	**0.02**	**0.19 (0.05–0.66)**	**0.01**
**Distance, 15-min drive or less as reference**				
16–30-min drive	0.87 (0.45–1.70)	0.69	0.69 (0.29–1.67)	0.42
>30-min drive	1.08 (0.48–2.42)	0.86	0.63 (0.20–2.03)	0.44
Flight	1.30 (0.53–3.17)	0.57	0.39 (0.11–1.38)	0.15
**Cancer type, breast as reference**				
Gastrointestinal	0.92 (0.46–1.84)	0.81	0.90 (0.27–3.05)	0.87
Hematopoietic	**2.87 (1.11–7.41)**	**0.03**	**4.99 (1.13–22.08)**	**0.03**
Genitourinary	1.78 (0.65–4.86)	0.26	1.38 (0.24–7.83)	0.72
Lung and other	2.19 (0.82–5.82)	0.12	2.61 (0.65–10.54)	0.18
**Cancer stage, 0–2 as reference**				
Stage 3–4	1.25 (0.67–2.32)	0.49	1.14 (0.45–2.91)	0.78
I do not remember	1.14 (0.56–2.32)	0.73	0.74 (0.23–2.33)	0.61
**Video included**	1.37 (0.77–2.42)	0.29	2.09 (0.48–2.46)	0.84
**Overall quality**	**8.74 (4.55–16.81)**	**<0.001**	**13.96 (6.10–31.99)**	**<0.001**

Bold values denote statistical significance at the p < 0.05 level.

OR: Odds ratio.

## Discussion

In the authors' study, the majority of patients (65.6%) found the overall quality of their telehealth visit to be equivalent to or better than office visits. Satisfaction with telehealth was even higher among patients whose visit included video (71.9%). This acceptance of telehealth visits is similar to that seen in a study by Donelan *et al.* performed prior to the pandemic, which showed that 75.2% of patients found the quality of the video visit to be equivalent to or better than a face-to-face visit [14].

Preference for wanting some future visits to be telehealth was seen in only 57.1% of patients, a stark difference from that seen in studies reported prior to the pandemic. Among radiation oncology patients, Hamilton *et al.* found that 89.6% of survey respondents wanted all or some of their visits to take place via telehealth [15]. In a non-oncology setting, Polinski *et al.* reported an even stronger desire for future telehealth visits, with 98% of patients stating that they would definitely or probably use telehealth again [16]. Although these studies were performed in different medical specialties, this discrepancy in preference for future telehealth visits may be in part due to the abrupt shift to telehealth in the authors' patient population compared with the cohorts studied by Hamilton *et al.* and Polinski *et al.*, for which telehealth was an accepted practice.

Asian and Native Hawaiian patients were less likely to desire future telehealth visits than White patients. This racial difference persisted even after adjusting for other sociodemographic factors. However, the authors did not identify an association between race and the perceived quality of the telehealth visit compared with traditional office visits. In a study conducted on face-to-face visits, Palmer *et al.* demonstrated that Asian and Pacific Islander cancer patients report worse communication with their providers and lower quality of care and self-efficacy than Whites [17]. These racial disparities likely carry over into telehealth visits and may be magnified by the additional challenges that come with virtual visits. In addition, when interviewed about their telehealth perceptions, Native Hawaiians highlighted the importance of nonverbal communication and the need to develop the patient–physician relationship to overcome differences in culture and ways of conceptualizing health [13]. The abrupt adoption of telehealth visits as a result of the COVID-19 pandemic may have resulted in encounters with practitioners who were not adept at addressing the cultural needs of Native Hawaiian patients over virtual visits.

In this study, the authors observed a large loss in patient–provider personal connection in telehealth visits. Only a small proportion (4.7%) of patients rated their personal connection with their oncology provider via telehealth as better than that seen during an office visit, whereas exactly half of the patients felt that office visits were better. This loss in personal connection persisted when restricting the analysis to only patients with video visits (47.5%) or patients who identified as White (44.0%). Donelan *et al.* showed a better personal connection among their patients, with only 32.7% of patients feeling that office visits were better than video visits [14]. Of note, there are significant differences between our patient populations. Donelan *et al.* surveyed predominantly White patients who presented to psychiatry, neurology and cardiology appointments. The authors' population was racially heterogeneous and made up exclusively of cancer patients. It is conceivable that oncology patients may create high levels of expectation for their relationship with the oncology provider [18]. This personal connection may be diminished when the visit is conducted digitally. Furthermore, the loss of expected personal connection may disproportionately affect the preference for telehealth in Asian and Native Hawaiian and Pacific Islander cancer patients, who represented a large part of the authors' study population. The authors' findings support the hypothesis that the preference for telehealth in culturally diverse groups may be dependent on whether it can nurture the patient–provider relationship despite its potential for improving the access to and quality of health care [13].

The authors found that income level impacted the inclusion of video as part of the telehealth visit and the desire for future telehealth visits. Patients who had audio-only visits were more often of lower income and may not have had the resources required for video visits. Although video enhances the quality of the visit, audio-only visits remain an important option for patients with lower income or poor digital literacy. A multivariate analysis demonstrated that patients with lower income ($30,000–89,999) were more likely to want future telehealth visits compared with patients with an income ≥$90,000. This finding is in contrast to recent research in which patients with the lowest income were less likely to use telehealth during the pandemic [19,20]; the authors' study showed an increased preference for telehealth in only the middle income bracket. This may highlight the ability of telehealth to overcome barriers to healthcare access that working people in a lower income group more often face, such as transportation challenges, gaining approval for time off work and finding childcare.

Of the study population, 85.8% lived on Oahu and likely did not experience telehealth prior to the pandemic. Furthermore, 34.6% of the patients on Oahu lived within a 15-min drive of the provider's office, a situation in which the convenience of telehealth may be less pronounced. Although the patients lived in varying proximity to their provider, the authors' study demonstrated that distance was not a significant factor in patient satisfaction or preference for telehealth. By contrast, a study conducted on spine patients in Texas and Pennsylvania during the pandemic found any distance greater than 10 miles to be a significant predictor of a preference for telehealth [21]. This may reflect differences in patient demographics, disease type and geographical preferences for telehealth during the pandemic.

The authors' study was focused on oncology patients and revealed that patients with hematopoietic cancers were significantly more likely to prefer future telehealth visits than breast cancer patients. Hematopoietic cancer patients are often reviewing laboratory test results during visits and may not have significant findings on physical exam. Breast cancer patients, however, regularly receive breast examinations during their visits and would be missing a more notable part of their normal doctor visit if the visit occurred via telehealth. Many patients expressed this concern in the free text response, stating that they preferred office visits specifically for the breast examination. Laboratory and imaging studies for the patients in this study were done on an outpatient basis, usually at locations convenient for the patient, demonstrating the utility of telehealth beyond the COVID-19 pandemic to provide remote follow-up care. Others have similarly found that telehealth visits often suffice in meeting cancer patient needs without further in-person care [22,23]. Although the authors' study found that certain cancer patients were more or less likely to prefer telehealth, it is imperative that providers accommodate patient preferences while continuing to provide appropriate clinical care.

## Limitations

This study is limited by its relatively small number of participants. Furthermore, all subjects receive their care in cancer clinics in Hawaii, which could limit the generalizability of the authors' findings. However, selecting this cohort of patients provided the authors the ability to analyze a multiracial cancer population with a large number of Native Hawaiians and Pacific Islanders. Similar to other patient survey studies, the authors' findings are subject to recall bias, if patients did not accurately recall their telehealth experience, as well as social desirability bias, as patients often provide answers they think their physician would like to hear.

## Conclusion & future perspective

The perceived quality of the telehealth visits and the desire for future telehealth visits were not uniform across different patient populations. Asian and Native Hawaiian and Pacific Islander patients were less likely to desire future telehealth visits in comparison with Whites. During the time frame of this study, distance was not the main driving factor for the use of telehealth, and our study demonstrated that distance did not significantly influence telehealth satisfaction in oncology patients. On the contrary, patients with lower income or hematopoietic cancers, in which the physical exam was less pertinent to care, were more likely to prefer future telehealth visits. The inclusion of video significantly enhanced the quality of the visit. Telehealth is a powerful tool in expanding care to oncology patients, and our results may guide both oncology providers and policymakers in better implementing telehealth during and after the COVID-19 pandemic. The preference for telehealth will continue to grow as telehealth visits become more normalized in future generations. Further studies and interventions are needed to overcome racial disparities in telehealth.

**Summary points**

- The COVID-19 pandemic has rapidly accelerated the use of telehealth in oncology practice.
- This study aimed to assess the perception of telehealth visits among a multiracial cancer population for whom in-person visits were the standard of care prior to coronavirus disease 2019.
- Asian and Native Hawaiian and Pacific Islander patients were less likely to desire future telehealth visits in comparison with Whites.
- Patients with lower income or hematopoietic cancers were more likely to prefer future telehealth visits.
- The inclusion of video significantly enhanced the quality of the visit.
- Future studies aimed at overcoming these racial disparities are needed to provide equitable oncology care through telehealth.
